# Construction of PdCu Alloy Decorated on the N-Doped Carbon Aerogel as a Highly Active Electrocatalyst for Enhanced Oxygen Reduction Reaction

**DOI:** 10.3390/gels11030166

**Published:** 2025-02-26

**Authors:** Yangxin Bai, Wenke Hao, Aleeza Altaf, Jiaxin Lu, Liu Liu, Chuanyong Zhu, Xindi Gu, Xiaodong Wu, Xiaodong Shen, Sheng Cui, Xiangbao Chen

**Affiliations:** 1 College of Materials Science and Engineering, Nanjing Tech University, Nanjing 210009, China; 18239945883@88.com (Y.B.); 18953496631@163.com (W.H.); aleezyaltaf1@gmail.com (A.A.); lujiaxin128@163.com (J.L.); liuliu6@njtech.edu.cn (L.L.); guxindi123456789@163.com (X.G.); xdshen@njtech.edu.cn (X.S.); scui@njtech.edu.cn (S.C.); 2 Jiangsu Collaborative Innovation Center for Advanced Inorganic Function Composites, Nanjing Tech University, Nanjing 211816, China; 3 College of New Energy, China University of Petroleum (East China), Qingdao 266580, China; cyzhu@upc.edu.cn; 4 AECC Beijing Institute of Aeronautical Materials, Beijing 100095, China; 15150675713@126.com

**Keywords:** aerogel, electrocatalyst, alloy, oxygen reduction reaction

## Abstract

Fuel cells/zinc–air cells represent a transformative technology for clean energy conversion, offering substantial environmental benefits and exceptional theoretical efficiency. However, the high cost and limited durability of platinum-based catalysts for the sluggish oxygen reduction reaction (ORR) at the cathode severely restrict their scalability and practical application. To address these critical challenges, this study explores a groundbreaking approach to developing ORR catalysts with enhanced performance and reduced costs. We present a novel Pd_3_Cu alloy, innovatively modified with N-doped carbon aerogels, synthesized via a simple self-assembly and freeze-drying method. The three-dimensional carbon aerogel-based porous structures provide diffusion channels for oxygen molecules, excellent electrical conductivity, and abundant ORR reaction sites. The Pd_3_Cu@2NC-20% aerogel exhibits a remarkable enhancement in ORR activity, achieving a half-wave potential of 0.925 V, a limiting current density of 6.12 mA/cm^2^, and excellent long-term stability. Density functional theory (DFT) calculations reveal that electrons tend to transfer from the Pd atoms to the neighboring *O, leading to an increase in the negative charge around the *O. This, in turn, weakens the interaction between the catalyst surface and the *O and optimizes the elementary steps of the ORR process.

## 1. Introduction

The development of environmentally sustainable and clean energy systems has become urgent due to the increasing environmental pollution and the crisis of traditional energy sources [1]. In today’s society, advanced energy systems require a continuous supply of sustainable and environmentally friendly energy sources, such as solar, wind, tidal, and biomass [2]. Nevertheless, the irregular spatial and temporal distribution of these clean energy sources has propelled the expedited development of sophisticated energy conversion devices and storage technologies, such as proton electrolyte membrane fuel cells (PEMFCs)/zinc–air cells [3,4]. Notably, the cathodic redox reaction, named oxygen reduction reaction (ORR) within PEMFCs, characterized by a complex and sluggish four-electron–proton-coupled transfer process with challenging reaction selectivity, represents the rate-limiting step in electricity generation. Consequently, the design of highly efficient and stable ORR electrocatalysts, aimed at mitigating the impediment of sluggish reaction kinetics, constitutes a pivotal aspect in advancing the development of PEMFCs [5,6].

Currently, precious-metal-based materials such as Pt, Au, Rh, and Pd have become leading electrocatalysts [7,8,9,10]. Among these, Pd has attracted wide attention due to its advantages of high activity, good selectivity, and mild reaction conditions [11,12]. Many strategies have been developed, and extensive efforts have been devoted to enhancing the ORR activity and durability of Pd-based nanocatalysts [13,14,15]. However, in the context of Pd nanocatalysts supported on carbon materials, the high surface energy of Pd nanoparticles, which serve as catalytic active centers, poses a challenge. At present, Pt-based noble metals and their alloys have been demonstrated to be excellent electrocatalysts for ORR in acid media [16,17]. This high energy predisposes them to agglomeration and deactivation during catalyst preparation and subsequent catalytic reactions, thereby compromising catalytic activity, selectivity, and the overall lifetime of the catalyst. To address this issue, enhancing the interaction between Pd nanoparticles and carbon supports through surface modification or doping of carbon materials has been explored. These strategies aim to improve the physical and chemical properties of the carbon support, leading to enhanced dispersion of the active Pd component and improved stability of the catalyst. Ultimately, this results in superior catalytic activity and selectivity, crucial for various applications in catalysis [18]. In particular, the N atom possesses a neighboring position to the C atom in the periodic table and features an additional electron in its outermost shell compared to carbon [19]. This introduction of an electron-rich element enhances the electron transport properties and chemical reactivity of carbon materials, leading to modifications in their energy band structure and physicochemical properties. As catalyst support, N-doped carbon (NC) material effectively mitigates the agglomeration of metal nanoparticles and fosters their uniform loading and dispersion on the surface, ultimately improving the catalytic activity and cyclic stability of the synthesized catalyst [20,21]. Lin et al. [22] proposed a pyrolytic approach to integrating atomically dispersed Fe sites with Pd nanocrystals embedded within NC nanoribbons. The electronic structure tuning of Pd by Fe-NC single atoms, in conjunction with the porous and doped characteristics of the NC framework, significantly reduced reaction barriers and drastically accelerated the ORR kinetics. The use of low-dimensional nanoribbons/sheets as catalyst supports is often limited by their low specific surface area, insufficient exposure of active sites, and tendency to stack. In contrast, incorporating porous or mesoporous carbon as the catalyst support provides several advantages, including a significantly higher specific surface area, a well-developed pore structure, excellent stability, and strong resistance to high temperatures and acidic or basic environments. These properties help maintain the original catalytic activity of the metal component while enhancing its interaction with the support [23,24]. Furthermore, the incorporation of nitrogen atoms into the carbon skeleton effectively modulates the physical and chemical properties of the material, its electron transport capacity, and the ligand metal charge density. This ultimately leads to the optimization of the ORR performance. Park et al. [25] developed a “raisin-bread”-like electrocatalyst consisting of cobalt nitride nanoparticles covered with a thin cobalt oxynitride layer and deposited on porous carbon aerogel. This electrocatalyst exhibited significant bifunctional activity for both the ORR and the OER in rechargeable zinc–air battery systems.

In this work, Pd is selected as the main catalytic component, based on the analysis results of ORR volcano maps. Cu is cleverly introduced in accordance with the Sabatier principle, aiming to fine-tune the adsorption energy of the intermediate and achieve an ideal binding strength that is neither too strong nor too weak. To prevent the agglomeration of metal particles, they are dispersed evenly onto the carbon matrix, and N atoms are further introduced to fine-tune the electron distribution density. This series of measures not only effectively reduces costs but also significantly improves catalytic efficiency. We prepared a new type of Pd_3_Cu alloy anchored in porous NC aerogel electrocatalyst by sol-gel and freeze-drying technology; the resulting aerogel catalyst possesses a distinctive “pearl chain” morphology, wherein as the extent of NC modification increases, the aerogel particles exhibit a reduction in size and a concomitant refinement in the internal pore structure. Notably, in alkaline conditions, the Pd_3_Cu@2NC-20% electrocatalyst demonstrates exceptional ORR performance, characterized by a half-wave potential of 0.925 V and a limiting current density of 6.12 mA/cm^2^. These findings highlight the potential of this novel catalyst system for efficient electrochemical applications. Furthermore, the Pd_3_Cu@2NC-20% electrocatalyst exhibited robust ORR performance under acidic and neutral conditions, surpassing the control Pt/C catalyst in terms of higher half-wave potentials and limiting current densities. DFT calculations further uncovered that the transfer of electrons from Pd atoms to the adjacent *O enhances the negative charge surrounding the *O, subsequently reducing the interaction strength between the *O and the catalyst surface, leading to an optimization of the binding energy of the adsorbed oxygen intermediates. This work not only demonstrates a cost-effective pathway to highly efficient Pd-based ORR catalysts but also paves the way for scalable, durable fuel cell technologies that can meet the demands of sustainable energy solutions.

## 2. Results and Discussion

### 2.1. Chemical Composition and Structural Analysis

**Figure 1:**
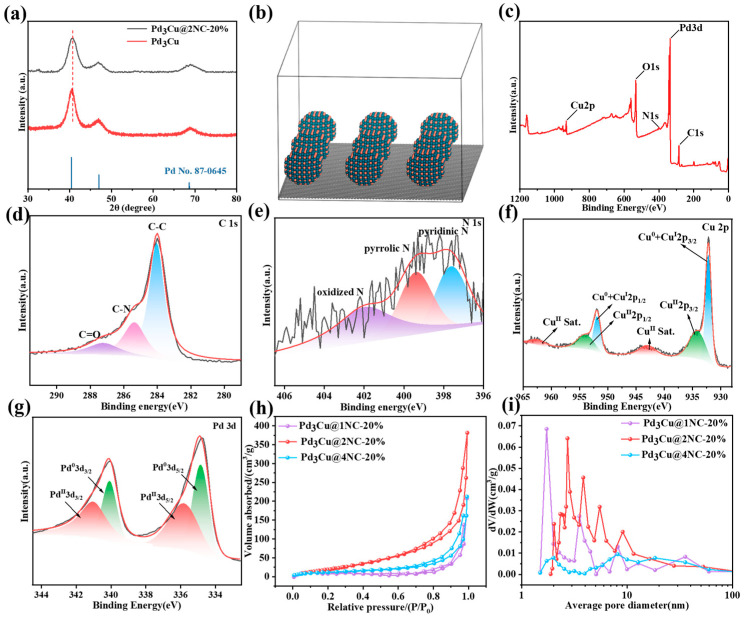
(**a**) XRD of Pd_3_Cu and Pd_3_Cu@ Pd_3_Cu@2NC-2% samples, (**b**) Atomic structure (green and orange represent the Pd and Cu atoms), (**c**) Survey scans, high-resolution XPS spectra, (**d**–**g**) The fitting curves of C peak, N peak, Cu2p peak, and Pd3d peak of Pd_3_Cu@2NC-20%, (**h**) N_2_ adsorption–desorption curves of three samples, and (**i**) BJH pore size distribution curves.

**Figure 2:**
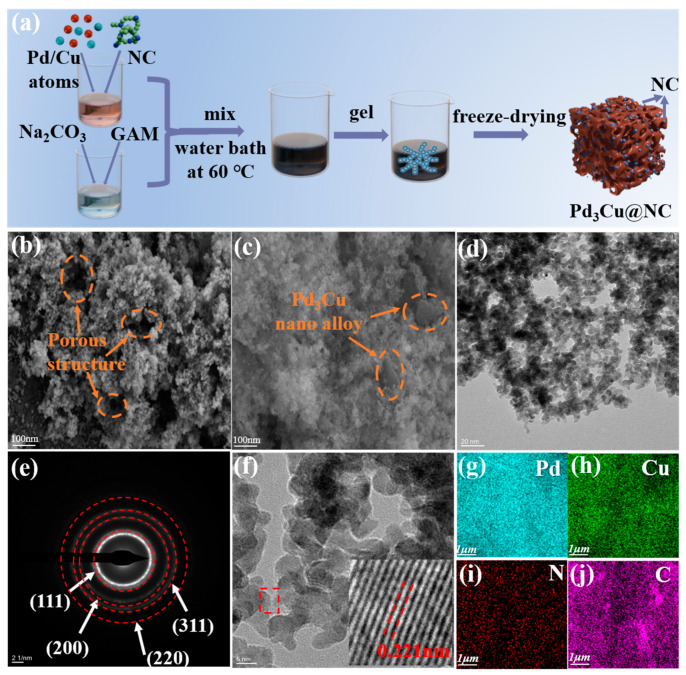
(**a**) Schematic illustration of the synthesis process of Pd_3_Cu@NC. SEM images of (**b**,**c**) Pd_3_Cu@2NC-20%, (**d**) TEM, (**e**) SAED, (**f**) HRTEM, and (**g**–**j**) EDX mapping images of the resulting Pd_3_Cu@2NC-20%.

**Figure 3:**
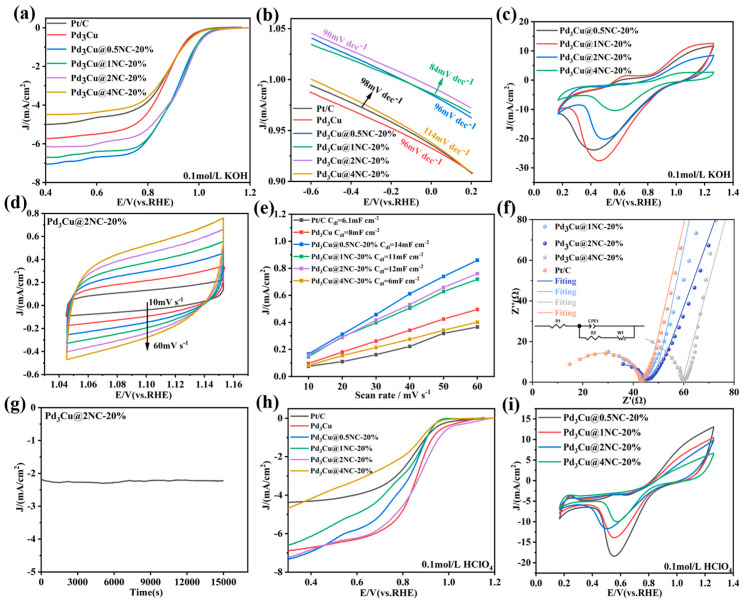
(**a**) LSV curves of Pt/C, Pd_3_Cu, Pd_3_Cu@0.5NC-20%, Pd_3_Cu@1NC-20%, Pd_3_Cu@2NC-20%, and Pd_3_Cu@4NC-20% in 1.0 M KOH electrolyte, (**b**) Tafel plots derived from the LSV curves, (**c**) CV curves in different O_2_-saturated solutions with a sweep rate of 50 mV s^−1^, (**d**) CV curves of Pd_3_Cu@2NC-20%, (**e**) Electrochemical double-layer capacitance tests of different samples, (**f**) EIS Nyquist plots of different samples, (**g**) Chronoamperometric stability test for the Pd_3_Cu@2NC-20% catalyst in 1.0 M KOH electrolyte, (**h**) LSV curves of different samples in acidic media, and (**i**) CV curves in different O_2_-saturated solutions with a sweep rate of 50 mV s^−1^.

X-Ray Powder Diffraction (XRD) analysis shows the crystalline structures of the Pd_3_Cu@2NC-20% and Pd_3_Cu samples (Figure 1a). The crystalline structure of these samples is demonstrated by their strong diffraction peaks. The observed diffraction peaks of the Pd_3_Cu@2NC-20% are very close to the standard reference pattern of Pd_3_Cu, confirming the efficient synthesis of the Pd_3_Cu phase. It is worth mentioning that, compared with Pd_3_Cu, the position of the Pd_3_Cu@2NC-20% peaks is slightly shifted, indicating that the crystal structure has a subtle change after loading the Pd_3_Cu in the NC matrix (atomic structure shown in Figure 1b). The XRD pattern of the Pd_3_Cu@2NC-20% shows distinct diffraction peaks at 2θ = 40.3, 46.9, and 69.1°, corresponding to the (111), (200), and (220) planes of an Fm-3m cubic structure. These peaks are consistent with the Pd_3_Cu alloy phase, confirming that the composite aerogel retains a Pd-dominated alloy structure [26]. The broadening and slight negative shift of the diffraction peaks relative to pure Pd metal indicate lattice expansion and reduced crystallinity, caused by the alloying of Pd atoms with Cu atoms. This lower crystallinity introduces structural features such as interfaces, defects, and distortions, which contribute to an increase in active sites during electrocatalytic processes. X-ray photoelectron spectroscopy (XPS) is used to analyze the surface chemical composition and electronic states of the synthetic materials (Figure 1c), and the spectra show obvious peaks corresponding to the nuclear grade binding energies of the component elements, confirming the presence of Pd, Cu, N, O, and C in the samples. In the high-resolution C1s XPS spectrum (Figure 1d), the peaks at 284.0, 285.3, and 287.2 eV are attributed to C-C, C-N, and C=O, respectively [27] Meanwhile, the N 1s spectrum (Figure 1e) can be decoupled into three peaks including pyridinic N at 397.6 eV, pyrrolic N at 399.3eV, and oxidized N at 401.9 eV, respectively [28,29]. In addition, the high-resolution Cu 2p spectra of the Pd_3_Cu@2NC-20% aerogel show distinct spin–orbit splitting peaks (Figure 1f), where the peaks 932.2 and 951.5 eV come from Cu 2p3/2 and 2p1/2 of Cu(0)/Cu^+^ [30]. Moreover, the peaks at 933.9 and 954.3eV can be attributed to 2p3/2 and 2p1/2 of Cu^2+^. In addition, similar satellite peaks associated with photoelectron lines were observed. For high-resolution Pd 3d spectra, the absorption peaks near 335.12 eV and 340.37 eV belong to 3d5/2 and 3d3/2 of Pd(0), and the absorption peaks near 336.2 eV and 341.5 eV belong to 3d5/2 and 3d3/2 of Pd^2+^ [31,32]. These results provide strong evidence for the existence of Pd_3_Cu@2NC-20% surface oxidation of the gas coagulant. The nitrogen adsorption–desorption isotherms of Pd_3_Cu@1NC-20%, Pd_3_Cu@2NC-20%, and Pd_3_Cu@4NC-20% are shown in Figure 2h, and the relevant pore structure information is shown in Table S1 (Supporting Information). The three samples have characteristic IV isotherms, which indicates that they have a typical mesoporous three-dimensional network structure. When the relative pressure (P/P_0_) is as high as 0.99, no saturated adsorption platform is reached, indicating that there are large pores in all three samples. It is worth mentioning that the optimal Pd_3_Cu@2NC-20% exhibits a BET-specific surface area of 98 m^2^/g, which is significantly larger than Pd3Cu (44.350 m^2^/g), Pd_3_Cu@1NC-20% (30.881 m^2^/g), and Pd_3_Cu@4NC-20% (43.083 m^2^/g), and also larger than the metal aerogel reported in the literature [33,34,35]. Consistent with the Electrochemical Surface Area (ECSA) structure in Figure 3e, this facilitates exposure of the active site, thereby accelerating the rate of charge transfer and ORR activity.

### 2.2. Electrocatalyst Preparation and Microstructure Analysis

The Pd_3_Cu@NC samples are synthesized by assembling Pd and Cu atoms onto the porous surface of NC aerogel using the chemical reduction. The preparation steps are summarized in Figure 2a. The structure of Pd_3_Cu@NC can be controlled by varying the amount of NC added during the gelation process (e.g., 0.5 mg, 1 mg, 2 mg, and 4 mg). Details of the synthesis are provided in the experimental section. The morphology and structure of the synthesized samples are analyzed using SEM (Figure S1), and the Pd_3_Cu@2NC-20% image shows a nanostructured material with a well-defined porous structure (Figure 2b). This porous structure is crucial for boosting catalytic activity, enhancing mass transport, and improving the efficiency of adsorption-driven applications. Meanwhile, Figure 2c highlights the Pd_3_Cu nano alloy, which is stacked along the edges of the pores, and larger pores (over 100 nm) are also visible within the structure. The TEM (Transmission Electron Microscope) image in Figure 2d shows that the Pd_3_Cu@2NC-20% aerogel has uniformly dispersed nanoparticles within the carbon matrix. The selected area electron diffraction (SAED) pattern in Figure 2e reveals diffraction rings corresponding to the (111), (200), (220), and (311) planes of the Pd_3_Cu nanoparticles [36,37]. These rings are diffuse rather than sharp, confirming the polycrystalline nature of the sample. In the high-resolution HRTEM image in (Figure 2f), the larger image shows the nanoscale morphology, while the inset highlights lattice fringes with a measured interplanar spacing of 0.221 nm, indicative of a well-defined crystalline structure. These crystalline features are essential for improving the material’s catalytic and electronic properties. The elemental mapping images in Figure 2g–j confirm the uniform distribution of Pd, Cu, C, and N throughout the aerogel. The EDS spectra (Figure S2) show that the Pd/Cu molar ratio (Pd_3_Cu@2NC-20%) calculated from the weight ratio is 2.52:1, which is similar to the results of the experimental process.

### 2.3. ORR Performance Analysis

In order to evaluate the effect of N-doping levels on the catalytic activity of ORR catalysts, six different N-doped catalysts were loaded onto an RDE (Rotating Disk Electrode) and measured by LSV (Linear Sweep Voltammetry) at room temperature in O_2_-saturated 0.1M KOH solutions at a sweep speed of 10 mV/s (Figure S3). The results in Figure S3 show that the Pd_3_Cu@2NC-20% (0.925 V) exhibits the most prominent catalytic activity. Simultaneously, in order to further explore the influence of the amount of NC on the catalytic activity of the catalyst, four catalysts with different amounts of NC are tested under the same operating conditions as above, and the LSV comparison diagram is drawn. The LSV polarization curve shows (Figure 3a) that, with the increase of NC load, the ORR performance of the samples increases first and then decreases. Among them, the optimal sample, Pd_3_Cu@2NC-20%, has the highest ORR activity with a half-wave potential of 0.925 V and a limiting current density of 6.12mA/cm^2^. The synergistic effect of the Pd_3_Cu and NC aerogels is verified. In addition, Tafel slope plots (Figure 3b,c) are performed to further verify the excellent ORR activity of the resulting Pd_3_Cu@2NC-20% aerogel. The linear region of the Tafel plot is fitted to the Tafel equation (η = blogj + a, where B is the Tafel slope). The linear region of the Tafel plot is fitted to the Tafel equation (η = blogj + a, where B is the Tafel slope) [38]. The Tafel slope of Pd_3_Cu@2NC-20% is 90 mV/dec, which is significantly lower than that of Pt/C (98 mV/dec) Pd_3_Cu (96 mV/dec), Pd_3_Cu@0.5NC-20% (96 mV/dec), and Pd_3_Cu@4NC-20% (114 mV/dec). It is close to that of Pd_3_Cu@1NC-20%, indicating that Pd_3_Cu@2NC-20% has faster reaction kinetics. In addition, by evaluating ORR activity in an O_2_-saturated 0.1M KOH solution, the optimal sample in Figure 3c has an obvious reduction method of Pd_3_Cu@2NC-20% at about 0.493 V, realizing its high ORR activity. The C_dl_ is obtained by CV at different scanning rates (Figure 3d), and the CV curves of other catalysts are shown in Figure S4. The Pd_3_Cu@2NC-20% is 12 mF/cm^2^ (Figure 3e), which is significantly larger than Pt/C (6.1 mF/cm^2^), Pd_3_Cu (8.0 mF/cm^2^), and Pd_3_Cu@1NC-20% (11 mF/cm^2^), and Pd_3_Cu@4NC-20% (6.0 mF/cm^2^) is close to Pd_3_Cu@0.5NC-20% (14 mF/cm^2^), which indicates that the large ECSA shown by the Pd_3_Cu@2NC-20% aerogel is rather consistent with the LSV polarization curve. In addition, the EIS results show (Figure 3f) that the best Pd_3_Cu@2NC-20% has a small charge transfer resistance, which is conducive to rapid electron transfer at the interface between the catalyst and the electrode [39]. In addition, the chronoamperometric method is tested, and the current density attenuation is negligible after 15,000 s, further demonstrating its excellent ORR activity and stability. Notably, Pd_3_Cu@2NC-20% also has excellent ORR activity (0.85V) under acidic conditions (Figure 3h,i). In addition, the Pd_3_Cu@2NC-20% aerogel catalyst is compared with other emerging catalysts. The results show that the Pd_3_Cu@2NC-20% ORR electrocatalytic performance reported in this paper is superior to most of the reported literature and is comparable to the results published in top journals [40,41,42,43,44,45,46,47,48,49,50,51,52] (Figure S5 and Table S2).

**Figure 4:**
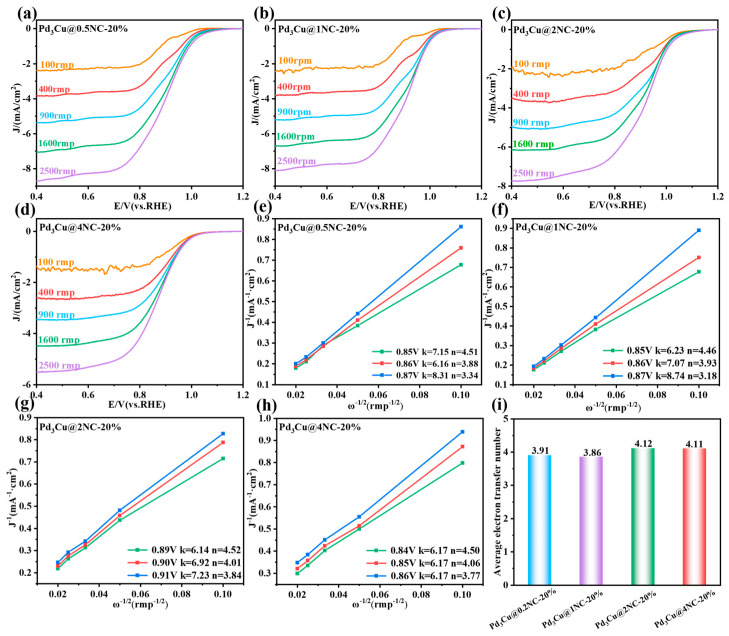
(**a**–**d**) ORR LSV curves of Pd_3_Cu@0.5NC-20%, Pd_3_Cu@1NC-20%, Pd_3_Cu@2NC-20%, and Pd_3_Cu@4NC-20% performed in O_2_-saturated 0.1 M KOH solution from 0.4 V to 1.2 V vs RHE at a scanning rate of 10 mV⋅s^−1^ with different rotation speeds, (**e**–**h**) Koutecky–Levich plots under different applied potentials for calculating the electron transfer number, (**i**) The histogram of average electron transfer numbers for different samples.

The ORR kinetics of Pd_3_Cu@NC catalysts were revealed using RDE technology, and the results are shown in Figure 4. Meanwhile, it can be seen that due to the promotion of mass transfer, the limiting current density becomes more negative as the speed increases [53,54]. The LSV curves (Figure 4a–d) show a clear dependence of the current density on the rotation speed of the electrode, highlighting the critical role of oxygen diffusion in the ORR process. The increase in limiting current density with rotation speed follows the K-L equation, which describes the linear dependence of current density on the square root of the rotation speed under mass transport control. To accurately measure the electron transfer number of different aerogel samples, we select three different applied potentials for calculation. As shown in Figure 4e–h, the slopes derived at these different applied potentials are very close, resulting in similar values for the derived electron transfer numbers. Additionally, we plotted histograms that show the number of electron transfers for different aerogel samples at various applied potentials (Figure 4i). It was found that for the optimal Pd_3_Cu@2NC-20% aerogel, the average electron transfer number is 4.12, which is quite close to 4.0. Furthermore, the mean electron transfer numbers for the original Pd_3_Cu@0.2NC-20%, Pd_3_Cu@1NC-20%, and Pd_3_Cu@4NC-20% are 3.91, 3.86, and 4.11, respectively. These results indicate that the electron transfer number for all four prepared catalysts is close to four suggesting that the ORR process of the prepared aerogels is a typical four-electron reaction, with water as the product. The rotating ring disk electrode (RRDE) test is used to further evaluate the electron transfer number and hydrogen peroxide yield. The Pd_3_Cu@1NC-20%, Pd_3_Cu@2NC-20%, and Pd_3_Cu@4NC-20% all underwent four-electron transfer reactions, with little hydrogen peroxide detected on the ring electrode. Specifically, the electron transfer number for Pd_3_Cu@2NC-20% ranged from 3.96 to 3.98, and the hydrogen peroxide yield is between 2% and 3%. These results further confirm that Pd_3_Cu@2NC-20% undergoes a four-electron transfer process with water as the product, and the high selectivity of the catalytic products favors its application in fuel cells, metal-air batteries, and electrochemical energy conversion devices (Figure S6).

### 2.4. OER Mechanism Analysis

**Figure 5:**
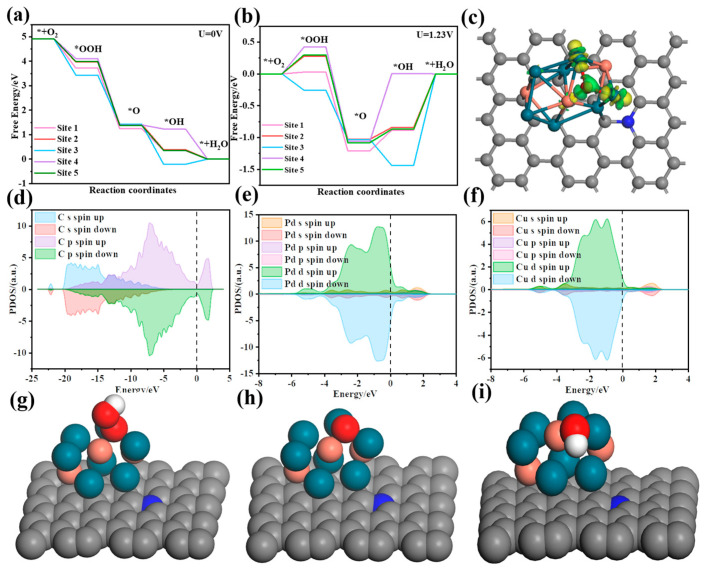
DFT calculations for the Gibbs free energy of the four ORR elementary steps for different kinds of reaction sites and structures at (**a**) U = 0 V and (**b**) U = 1.23 V, (**c**) Electron density difference, PDOS of (**d**) C, (**e**) Pd, and (**f**) Cu of the geometry-optimized configuration of the Pd_3_Cu@NC with the intermediate *O, and (**g**–**i**) Geometry-optimized configurations of *OOH, *O, and *OH for Site 2.

To further explore the ORR mechanism of the Pd_3_Cu@NC catalyst, we performed DFT calculations. The catalytic process of the Pd_3_Cu@NC catalyst is composed of four consecutive steps of coupled transfers of protons and electrons with *OOH, *O, and *OH intermediates, with oxygen adsorption being a prerequisite for the subsequent four steps, as our previous experimental validation confirmed [55,56]. For this purpose, five models with different active sites were constructed (Figure 5g–i and Figures S7–S10). Free energy diagrams for the ORR at different reaction sites (Site 1, Site 2, Site 3, Site 4, and Site 5) under U = 0 V (Figure 5a) and U = 1.23 V (Figure 5b) are computed using the CHE (Computational Hydrogen Electrode) method. The CHE method is used to calculate the Gibbs free energy curves of the four basic steps of different models (Figure 5a,b), and the relative Gibbs free energy data are shown in Table 1. It is well known that the rate-determining step and theoretical overpotential of the ORR process correspond to the step with the lowest downhill energy. It can be seen that for Site4 and Site5 in Pd_3_Cu@2NC-20%, the rate-determining steps are ΔG3 and ΔG4, respectively, and the theoretical overpotential are 1.069 and 0.87 eV, respectively. For Site1–Site3, the rate-determining step is ΔG4, and the theoretical overpotential from *OH to the exposed surface is 0.884, 0.843, and 1.411 eV, respectively. It is worth mentioning that ΔG4 is even positive in Site3, which indicates that the surface binding of *OOH and *O of the catalyst is too strong and impeding ORR activity. In summary, for the Pd_3_Cu@2NC-20% aerogel electrocatalyst, the minimum downhill step of Site2 is −0.387, so it has the smallest theoretical overpotential (0.843 eV) among the five active sites, which is consistent with the LSV results in Figure 3a. At the same time, the PDOS (Projected Density of State) diagram of the C atom in the catalyst system (Figure 5d) shows the contribution of s and p orbitals to the spin-up and spin-down electrons. The PDOS diagram shows that the p orbital of C makes a significant contribution to the electronic states near the Fermi level, which plays a leading role in determining the catalytic activity of the material. The PDOS of the Pd and Cu atoms (Figure 5e,f) show that the d orbitals are major contributors to the electronic states near the Fermi level, underscoring their critical role in determining the catalytic properties of Pd-based materials. As can be seen in Figure 5g–i, electrons tend to transfer from Pd atoms to neighboring *O, leading to an increase in negative charge around the *O. This, in turn, weakens the interaction between the catalyst surface and *O, further verifying the mechanism of the greatly enhanced ORR activity.

## 3. Conclusions

In summary, we successfully synthesized the Pd_3_Cu@NC electrocatalyst through a mild reduction process using glyoxylic acid monohydrate combined with a freeze-drying technique. The resulting aerogel catalyst exhibits a distinctive “pearl chain” structure with abundant porosity and a high BET-specific surface area (96.098 m^2^/g). The aerogel composite exhibits a significant improvement in ORR activity, attaining a half-wave potential of 0.925 V, a limiting current density of 6.12 mA/cm^2^, and outstanding long-term stability. Importantly, NC significantly reduces the use of precious metals while enhancing the ORR performance of the aerogel catalysts, effectively lowering production costs. DFT calculations further revealed that electron transfer from Pd atoms to the neighboring *O increases the negative charge around *O, thereby weakening the interaction between *O and the catalyst surface, optimizing the binding energy of the adsorbed O intermediates. Overall, this work provides valuable insights for the development of cost-effective noble metal-based aerogel electrocatalysts synthesized under mild conditions, showcasing strong potential for industrial applications.

## 4. Materials and Methods

### 4.1. Materials

Resorcinol (C_6_H_6_O_2_, 99.5%) and urea (CO(NH_4_)_2_) were purchased from Sinopharm Chemical Reagent Co., Ltd., Shanghai, China. Sodium carbonate (Na_2_CO_3_, 99.8%) was purchased from Shanghai Lingfeng Chemical Reagent Co., Ltd., Shanghai, China and palladium chloride (PdCl_2_, 98%) was purchased from Jiangsu YiKang Biological Medicine Research and Development Co., Ltd., Nantong, China. Formaldehyde, copper (II) chloride dihydrate (CuCl_2_·2H_2_O, 99.8%), and glyoxalic acid monohydrate (CHOCOOH·H_2_O, 99.8%) were bought from Shanghai McLean Biochemical Technology Co., Ltd., Shanghai, China. The commercial Pt/C catalyst (20 wt% Pt loading) was obtained from Strem Chemicals, Boston, MA, USA. Anhydrous ethanol (EtOH, 99.7%) was provided by Wuxi Yasheng Chemical Reagent Co., Ltd., Wuxi, China and deionized water (H_2_O, 99.9%) was from a Direct-Q system with a resistivity of 18.2 MΩ/cm. All the chemicals and reagents were used as received without further purification.

### 4.2. Method

#### 4.2.1. Synthesis of N-Doped Carbon Aerogel

The NC aerogel was prepared using the sol-gel method. For a typical synthetic route, 450 mg of resorcinol (R) was dissolved with 107 mg of urea and 0.8 mL of formaldehyde (F) in 3.4 mL of deionized water, and the mixture was magnetically stirred for 10 min. Sodium carbonate (Na_2_CO_3_) was then added to the uniform mixture, and the pH was adjusted to 7. The mixture was further stirred for 30 min. The resulting solution was then transferred to an oven maintained at 50 °C for gelation. The wet gel subsequently underwent a solvent exchange process, where it was soaked in an anhydrous ethanol bath for 3 days to effectively replace the water and reaction byproducts within the gel pores. After the aging and solvent exchange, the wet gel was dried using supercritical CO_2_ to obtain RF aerogel. In order to achieve N-doping, the RF aerogel was heated to 900 °C at a rate of 3 °C/min in an N_2_ atmosphere and held at that temperature for 2 h. The NC aerogel with 20% N-doping was prepared through this process. According to a similar scheme, carbon aerogels with different N-doping levels were synthesized by adjusting the amount of urea in the initial mixture.

#### 4.2.2. Synthesis of Pd_3_Cu@NC Aerogel

The Pd_3_Cu was prepared using our previously reported method [26]. In short, solution A was obtained by dissolving PdCl_2_ (3 mM), CuCl_2_ (1 mM), and 2 mg NC in 10 mL of deionized water. Solution B was obtained by dissolving 371 mg of Na_2_CO_3_ and 46 mg of glyoxylic acid monohydrate (GAM) in 10 mL of deionized water. Solution B was poured into solution A and stirred at 60 °C in a water bath until the mixed solution turned black. The mixed solution was then placed in a 50 °C oven for gel treatment, and the wet gel was replaced with water for 3 days. Finally, the gel was dried by a freeze-drying technique to obtain Pd_3_Cu/NC aerogel, which can be denoted as Pd_3_Cu@aNC-b (a = 2, b = 20%), where a represents the mass of N-doped carbon aerogel, and b represents the percentage of N-doped. The gel was prepared by the same method, and the mass of NC was changed to 0.5, 1, and 4 mg, which were named Pd_3_Cu@0.5NC-20%, Pd_3_Cu@1NC-20%, and PdCu@4NC-20%.

#### 4.2.3. Characterizations

The X-ray diffraction (XRD), D8 (Bruker, Bremen, Germany), patterns of each nanostructure were tested using a powder X-ray diffractometry with Cu Kα (λ = 0.15406 nm) radiation in the 2θ range of 30 to 80°, operating voltage at 40 kV, 40 mA, and scanning rate of 10°/min. The surface chemical group of the resulting samples was analyzed by an X-ray photoelectron spectroscopy (XPS), ESCALAB 250XI (Thermo Fisher Scientific, Waltham, MA, USA), analyzer. Due to the electrical conductivity of the metal-based NC aerogels, the microstructures and morphologies of the samples were investigated by scanning electron microscopy (SEM) using a Sigma 300 (Zeiss, Oberkochen, Germany) field emission scanning electron microscope. The N_2_ adsorption–desorption pore structure analysis, including BET-specific surface area, Barrett–Joyner–Halenda (BJH) pore size distribution, and BJH adsorption pore volume, was performed on a Micrometrics ASAP2020 (Gold APP Instruments Co., Beijing, China) surface area and pore distribution analyzer. Vacuum degassing was carried out before the BET analysis, and the pretreatment condition was 120 °C with a residence time of 6 h. The microstructure and crystal structure of the resulting aerogels were further tested using FEI Talos F200S (Thermo Fisher Scientific, Waltham, MA, USA) Transmission Electron Microscopy (TEM) equipment.

#### 4.2.4. Electrochemical Measurements

All the electrochemical data were measured by an electrochemical workstation (CS2350), and the ORR measurements were performed using a conventional three-electrode system. A rotating disk electrode (RDE) with a diameter of 5 mm, a platinum sheet, and an Ag/AgCl electrode containing a saturated aqueous potassium chloride were chosen as the working electrode, counter electrode, and reference electrode, respectively. The catalyst ink was prepared by mixing 4 mg of catalyst, 1 mL of EtOH, and 40 μL of 5 wt% Nafion, and sonicated for 1 h. The prepared ink was drop-coated on the RDE and evaporated, with the catalyst loading of 600 μg/cm^2^. After electrochemical conditioning by 10 cyclic voltammetry (CV) scans reached a stable state, the linear sweep voltammetry (LSV) curves were recorded at a scan rate of 10 mV/s. In order to deduct the background currents, the CV and LSV curves for the samples were tested under a nitrogen atmosphere with the same parameters. The ORR experiments were performed in various electrolyte solutions with different pH values (0.1 M KOH, phosphate buffer, and 0.1 M HClO_4_ aqueous solution) at room temperature. Initial voltage (V) = 0, high frequency (Hz) = 100, 000, low frequency (Hz) = 0.01, amplitude (V) = 0.005, and quiet time (s) = 2 were the experimental conditions of the electrochemical impedance spectroscopy (EIS). Based on the Nernst equation, the reference potential is calibrated to a reversible hydrogen electrode (RHE) [26]:

(1) $$V_{RHE}=V_{Hg/HgO}+0.0592\times pH+0.098$$

The RDE test was performed under different rotating speeds, and the electron transfer number was evaluated via the Koutecky–Levich (K-L) equation [57]:

(2) $$\frac{1}{J}=\frac{1}{J_{L}}+\frac{1}{J_{K}}$$

(3) $$J_{L}=0.2nFD^{\frac{2}{3}}v^{-\frac{1}{6}}W^{\frac{1}{2}}C$$

where J represents the measured current density, while J_k_ and J_L_ are the kinetic and limiting current density, ω is the disc rotation angular velocity of the disk, n represents the calculated electron transfer number in ORR, and F is the Faraday constant (F = 96,485 C·mol^−1^), C is the bulk concentration for O_2_ (1.2 × 10^−6^ mol·cm^−3^) dissolved in 0.1 m KOH solution, D is the diffusivity of O_2_ (1.9 × 10^−5^ cm^2^·s^−1^), and *ν* is the kinematic viscosity of electrolyte (0.01 cm^2^·s^−1^).

The rotating ring–disk electrode (RRDE) was operated at a rotation speed of 1600 rpm and a scan rate of 10 mV·s^−1^ to measure the disk current (Id) and the ring current (Ir). The hydrogen peroxide yield (H_2_O_2_%) and the electron transfer number (n) were derived using the following equations [28]:

(4) $$H_{2}O_{2}\left(\%\right)=\frac{200\times I_{r}}{\left(N\times I_{d}\right)+I_{r}}$$

(5) $$n=\frac{4\times I_{d}}{I_{d}+I_{r}/N}$$

#### 4.2.5. Theoretical Calculations Details

All the spin unrestricted density functional theory (DFT) calculations in this study, including structure optimizations and electronic structures, were performed using the DMol3 module of the Materials Studio 8.0 software package. The Perdew, Burke, and Ernzernhof functional (GGA-PBE), and the double-numerical basis set (DNP), were adopted to describe the electron exchange and correlation interactions. The effective core potential model was adopted to evaluate the metal relativistic effect for the core treatment, and smearing of 0.005 Ha was used for the orbital fractional occupation. Four models were considered in this study to reveal the real active sites for the greatly enhanced ORR activity. The optimal Pd_3_Cu@NC model was constructed using the following method. The graphene layer was used as the base, where one C atom was replaced by an N atom, and seven Pd atoms and three Cu atoms were constructed on the base, which was based on the SEM–EDS spectrum (close to Pd/Cu molar ratio as revealed by 2.52:1). A vacuum region of 15 Å thick was performed in the vertical direction to the model layer to eliminate the interaction between each slab and its images. A 4 × 4 × 1 supercell was further constructed with a lattice parameter of a = 9.84 Å and b = 11.00 Å, therefore obtaining the final Pd_3_Cu@NC model. The Brillouin zone was sampled with Monkhorst–Pack 4 × 4 × 1 k-point mesh for different models. During the DFT geometry optimization calculations, the bottom layer was fixed while the top two layers were fully allowed to relax to the ground state. Zero-point energy correction was included in all the reported energies. In addition, the geometry convergence tolerance was set to be 2.0 × 10^−3^ Hartree/Å, 1.0 × 10^−5^ Hartree, and 5.0 × 10^−3^ Å for maximum force, energy, and displacement, respectively. A 4 × 4 × 1 k-points mesh was also involved in the electronic structure calculations.

The computational hydrogen electrode (CHE) model was developed with Gibbs free energy calculations of each elementary step. In addition, zero point energy (ZPE) and entropy values of the adsorbed intermediates were obtained by the frequency calculation. A temperature of 298.15 K was used for all the calculations. The free energy of the O_2_ molecule was calculated by the following equation:

(6) $$G_{O_{2}}=2G_{H_{2}O}\;-\;2G_{H_{2}}+4.92$$

which is obtained from the standard redox potential of H_2_O/O_2_. The ORR process can be composed of four elementary steps as follows:

(7) $$*+O_{2}\left(g\right)+4H^{+}+4e^{-}\to *OOH+3H^{+}+3e^{-}$$

(8) $$*OOH+3H^{+}+3e^{-}\to *O+2H^{+}+2e^{-}+H_{2}O$$

(9) $$*O+2H^{+}+2e^{-}+H_{2}O\to *OH+H^{+}+e^{-}+H_{2}O$$

(10) $$*OH+H^{+}+e^{-}+H_{2}O\to *+2H_{2}O$$

where the * indicates the active site and *X represents the intermediates during the ORR process. The adsorption-free energy of the reaction intermediates was obtained via the following equations:

(11) $$∆G_{*O_{2}}=G_{*}+2G_{H_{2}\;}-\;2G_{H_{2}O}\;-\;4.92$$

(12) $$∆G_{*OOH}=G_{*OOH}\;-\;G_{*}+1.5G_{H_{2}}\;-\;2G_{H_{2}O}$$

(13) $$∆G_{*OH}\;-\;G_{*}+0.5G_{H_{2}}\;-\;G_{H_{2}O}$$

(14) $$∆G_{*O}=G_{*O}\;-\;G_{*}+G_{H_{2}}\;-\;G_{H_{2}O}$$

where G_*X_ represents the Gibbs free energy of the reaction intermediates, while G_*_ is the pristine catalyst surface. In addition, the Gibbs free energy of each step can be expressed as follows:

(15) $$∆G=∆E+∆ZPE\;-\;T∆S+∆G_{U}+pH*K_{B}T\;ln10$$

where ΔE, ΔZPE, and ΔS are the reaction energy, the change in zero point energy, and entropy between two steps.

(16) ∆Gu=eU

where e is the number of the transferred electrons and U is the applied potential vs. RHE. K_B_ is the Boltzmann constant, and it is worth mentioning that we take pH = 0 in our calculations for ORR under acidic conditions.

## Figures and Tables

**Table 1:** Gibbs free energy calculations of the elementary steps of the four models.

Samples	ΔG_OH*_	ΔG_O*_	ΔG_OOH*_	ΔG1	ΔG2	ΔG3	ΔG4
Site1	0.345	1.244	3.717	−1.202	−2.472	−0.898	−0.346
Site2	0.386	1.431	3.965	−0.954	−2.534	−1.044	−0.387
Site3	−0.211	1.429	3.432	−1.488	−2.003	−1.640	0.211
Site4	1.232	1.394	4.110	−0.809	−2.716	−0.161	−1.232
Site5	0.359	1.380	3.988	−0.932	−2.609	−1.020	−0.359

## Data Availability

The raw/processed data required to reproduce these findings cannot be shared at this time as the data also form part of an ongoing study.

## Supplementary Materials

The following supporting information can be downloaded at: https://www.mdpi.com/article/10.3390/gels11030166/s1, Figure S1. SEM images of (a,b) Pd_3_Cu@1NC-20%. Figure S2. EDS spectra of the Pd_3_Cu@2NC-20% aerogel. Figure S3. LSV curves of samples with different NC ratios at 0.1M KOH in an O_2_-saturated atmosphere at 1600rpm and a sweep speed of 10 mV/s. Figure S4. CV curves at different sweep speeds (a) Pd_3_Cu, (b) Pd_3_Cu@0.5NC-20%, (c) Pd_3_Cu@1NC-20%, (d) Pd_3_Cu@2NC-20%, and (e) Pd_3_Cu@4NC-20%. Figure S5. The comparison of ORR performance of as-prepared Pd3Cu@2NC-20% and other reported electrocatalysys in the literature. Figure S6. RRDE test of Pd3Cu@1NC-20%, Pd3Cu@2NC-20% and Pd3Cu@4NC-20%. Figure S7. (a–c) Top view of site1 adsorption states OOH, O, and OH; (d–f) Main view of site1 adsorption states OOH, O, and OH. Figure S8. (a–c) Top view of site1 adsorption states OOH, O, and OH; (d–f) Main view of site3 adsorption states OOH, O, and OH. Figure S9. (a–c) Top view of site1 adsorption states OOH, O, and OH; (d–f) Main view of site4 adsorption states OOH, O, and OH. Figure S10. (a–c) Top view of site1 adsorption states OOH, O, and OH; (d–f) Main view of site5 adsorption states OOH, O, and OH. Table S1. Pore structure data of both samples. Table S2. Comparison of ORR performance of the as-prepared Pd3Cu@2NC-20% sample in this work and other electrocatalysts reported in the literature.
