# Supplementary material for: Construction of PdCu Alloy Decorated on the N-Doped Carbon Aerogel as a Highly Active Electrocatalyst for Enhanced Oxygen Reduction Reaction
Source: Gels. 2025 Feb 26;11(3):166. doi: 10.3390/gels11030166 (PMC11942533; doi:10.3390/gels11030166)
Supplement: Supplementary file 1 [file gels-11-00166-s001.zip › gels-3440872-supplementary.pdf]

Supporting Information

Construction of PdCu alloy decorated on the N-doped carbon aerogel as a highly active electrocatalyst for enhanced oxygen reduction reaction

Yangxin Bai [1,2,†], Wenke Hao [1,2,†], Aleeza Altaf [1,2,†], Jiaxin Lu [1,2], Liu Liu [1,2], Chuanyong Zhu [3], Xindi Gu [1,2], Xiaodong Wu [1,2,*], Xiaodong Shen [1,2], Sheng Cui [1,2] and Xiangbao Chen [4]

1 College of Materials Science and Engineering, Nanjing Tech University, Nanjing 210009, China; 18239945883@88.com (Y.B.); 18953496631@163.com (W.H.); aleezyaltaf1@gmail.com (A.A.); lujiaxin128@163.com (J.L.); liuliu6@njtech.edu.cn (L.L.); guxindi123456789@163.com (X.G.); xdshen@njtech.edu.cn (X.S.); scui@njtech.edu.cn (S.C.)

2 Jiangsu Collaborative Innovation Center for Advanced Inorganic Function Composites, Nanjing Tech University, Nanjing 211816, China

3 College of New Energy, China University of Petroleum (East China), Qingdao 266580, China; cyzhu@upc.edu.cn

4 AECC Beijing Institute of Aeronautical Materials, Beijing 100095, China; 15150675713@126.com

* Correspondence: wuxiaodong@njtech.edu.cn

† These authors contributed equally to this work.

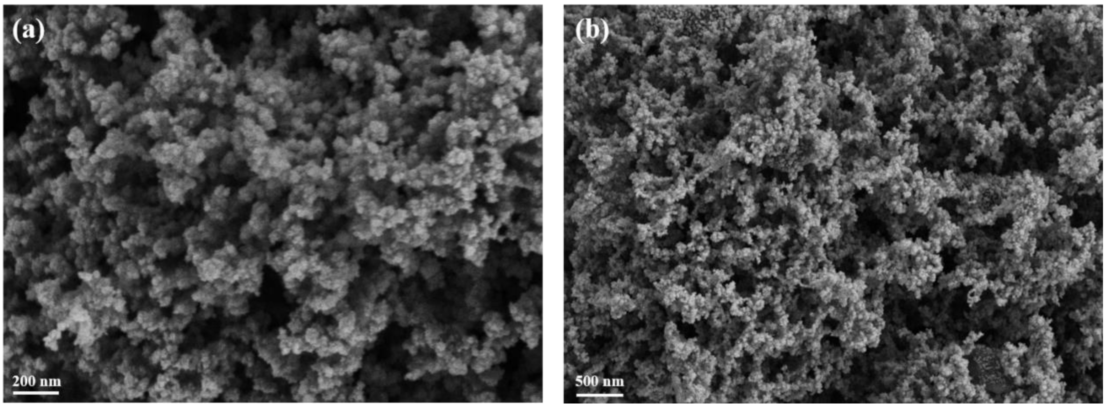

Figure S1 SEM images of (a-b) Pd_3Cu@1NC-20%.

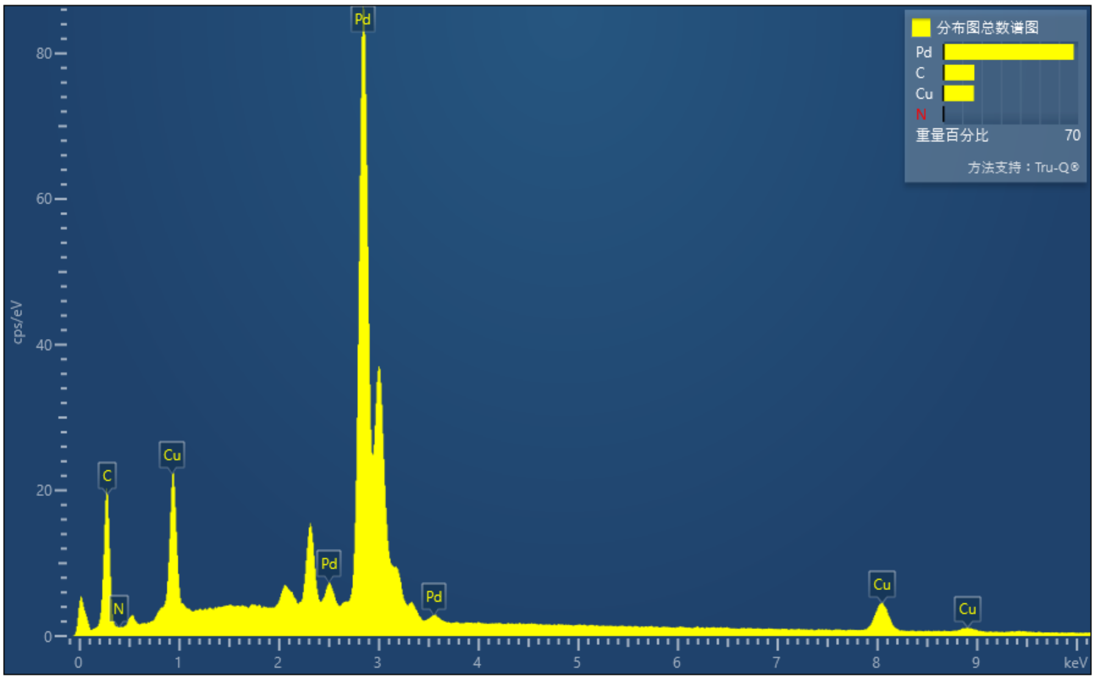

Figure S2 EDS spectra of the Pd3Cu@2NC-20% aerogel.

Table S1 Pore structure data of both samples.

**Sample**	**Specific Surface Areas ($m^2 \cdot g^{-1}$)**	**BJH adsorption average pore diameter (nm)**	**BJH adsorption Pore volume ($cm^3 \cdot g^{-1}$)**
Pd_3Cu@1NC-20%	30.881	38.955	0.3007
Pd_3Cu@2NC-20%	96.098	21.133	0.5077
Pd_3Cu@4NC-20%	43.083	27.894	0.3004
Pd_3Cu	44.350	8.770	0.0940

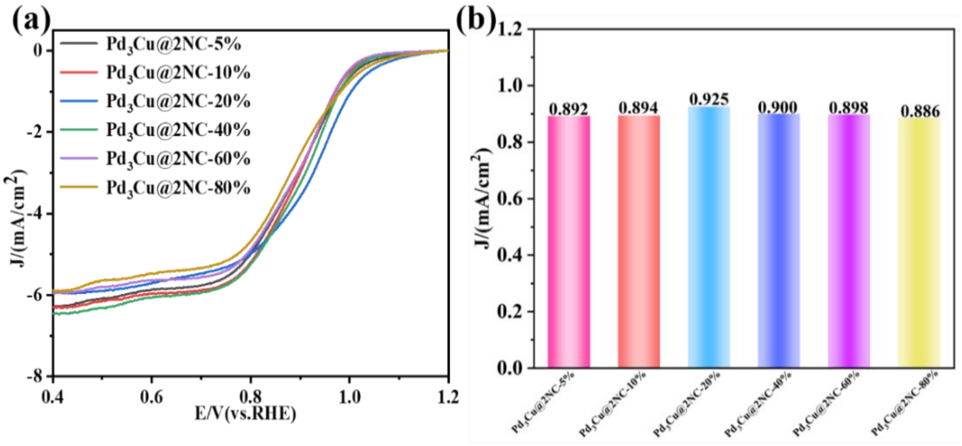

Figure S3 LSV curves of samples with different NC ratios at 0.1M KOH in an

O2-saturated atmosphere at 1600rpm and a sweep speed of 10mV/s.

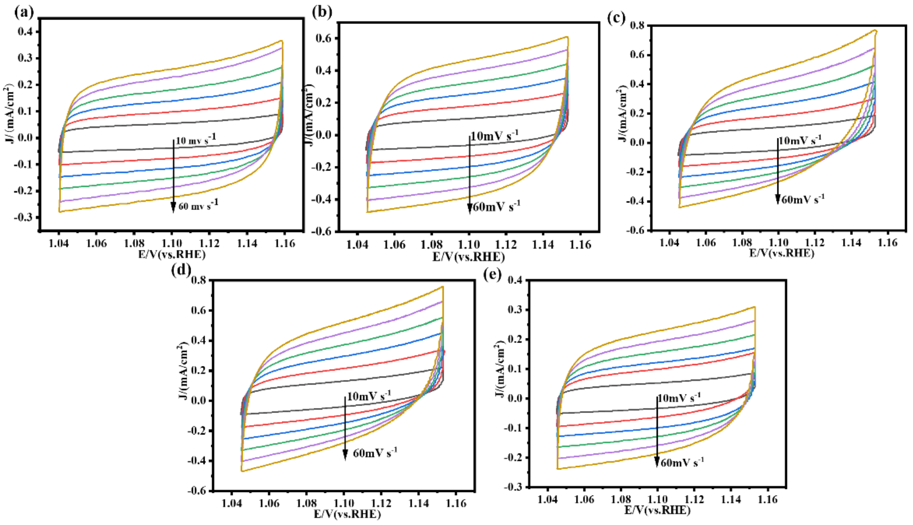

Figure S4 CV curves at different sweep speeds (a) Pd_3Cu, (b) Pd_3Cu@0.5NC-20%, (c) Pd_3Cu@1NC-20%, (d) Pd_3Cu@2NC-20%, and (e) Pd_3Cu@4NC-20%.

Table S2 Comparison of ORR performance of the as-prepared Pd3Cu@2NC-20% sample in this work and other electrocatalysts reported in the literature.

Catalyst	Half-wave potential (V)	Limiting current density (mA/cm^2)	Rference
PCF-FeTz-900	0.850	5.25	[1]
Co/$CoSe_2$@NSeC	0.865	6.00	[2]
$Co(OH)_2$/NCNT	0.920	5.10	[3]
Fe-Ni@N/C	0.877	5.25	[4]
Pd_3Cu	0.900	5.80	[5]
RuO_2/CMO	0.800	5.20	[6]
CoFe-NiFe@NCNT	0.850	5.14	[7]
Fe_2-$S1N_5N_5$/SNC	0.829	6.07	[8]
FeNi-NC@MWCNT	0.900	5.63	[9]
CoFe/CNT	0.895	5.45	[10]
Co-NrGO-SACs	0.840	5.90	[11]
Ncpor-Co	0.830	5.35	[12]
CoSAs-NPC	0.84	6.16	[13]

**This work**	**0.925**	**6.12**

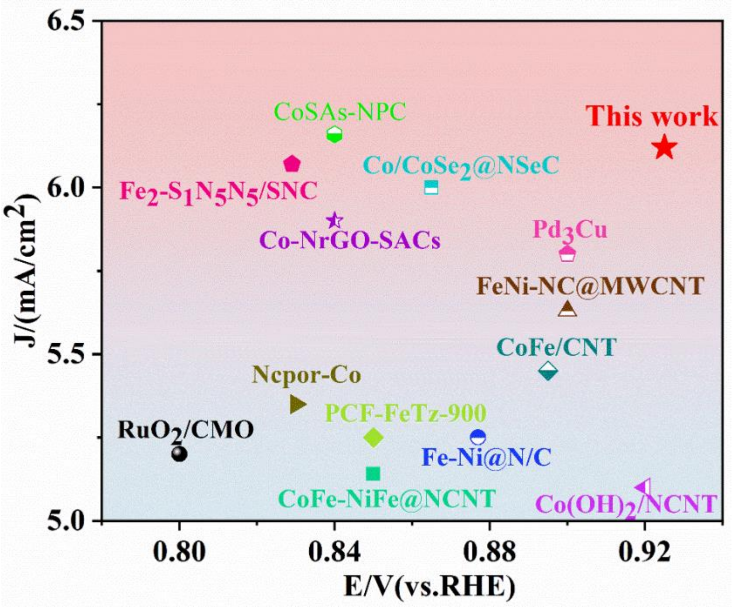

Figure S5. The comparison of ORR performance of as-prepared Pd3Cu@2NC-20% and other reported electrocatalysys in the literature.

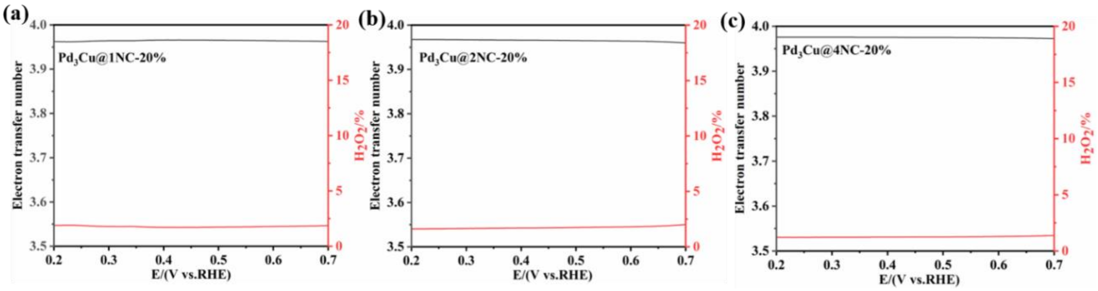

Figure S6. RRDE test of Pd3Cu@1NC-20%, Pd3Cu@2NC-20% and Pd3Cu@4NC-20%.

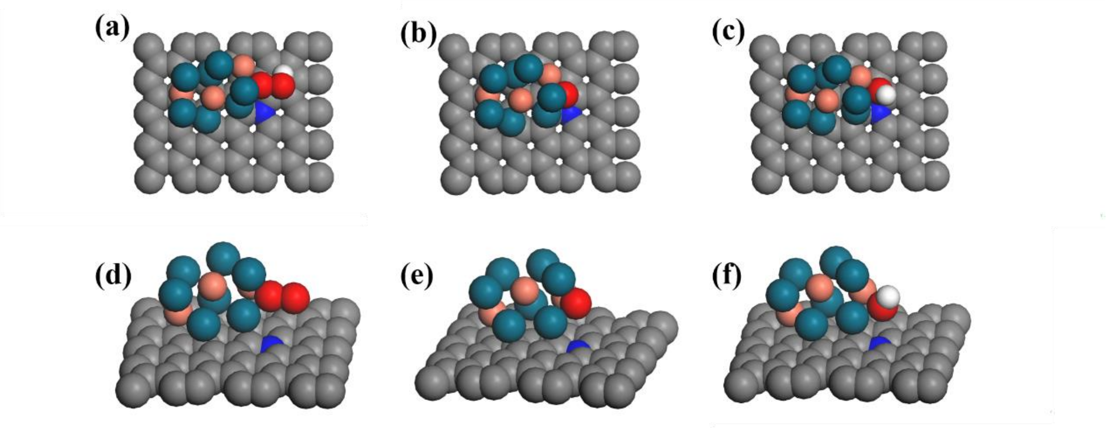

Figure S7 (a-c) Top view of site1 adsorption states OOH, O, and OH; (d-f) Main view of site1 adsorption states OOH, O, and OH.

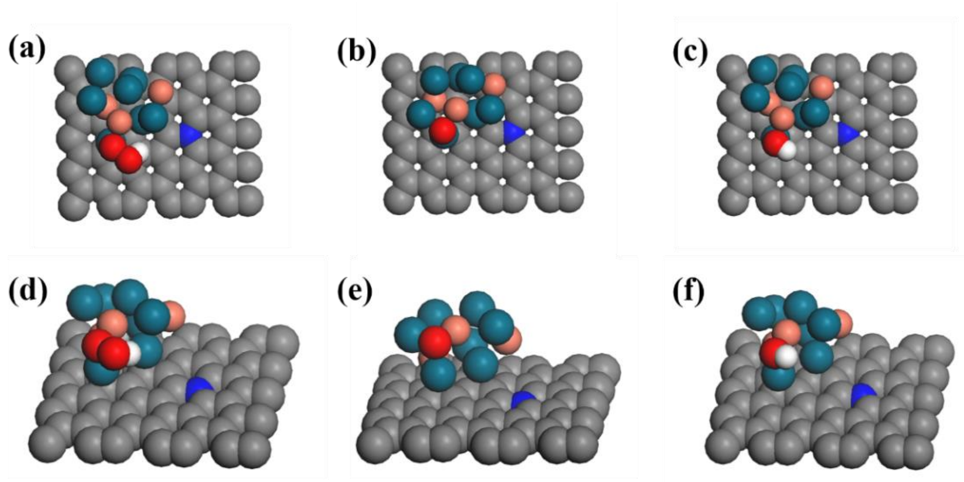

Figure S8 (a-c) Top view of site1 adsorption states OOH, O, and OH; (d-f) Main view of site3 adsorption states OOH, O, and OH.

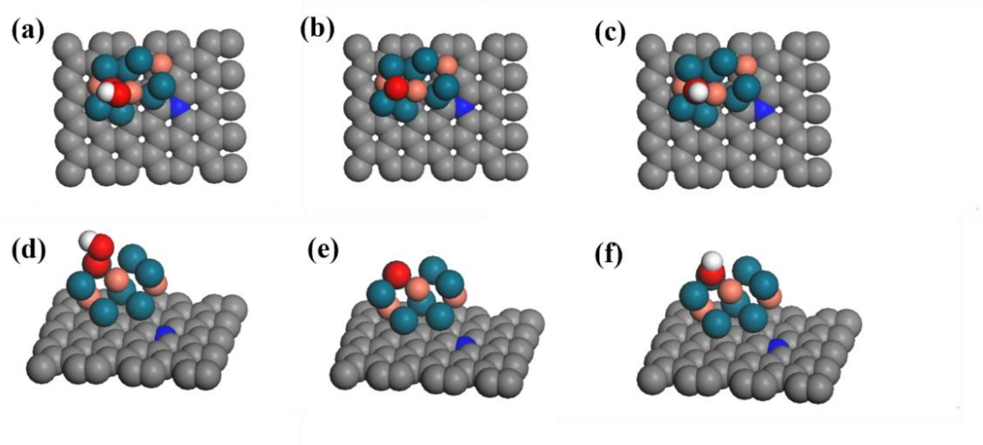

Figure S9 (a-c) Top view of site1 adsorption states OOH, O, and OH; (d-f) Main view of site4 adsorption states OOH, O, and OH.

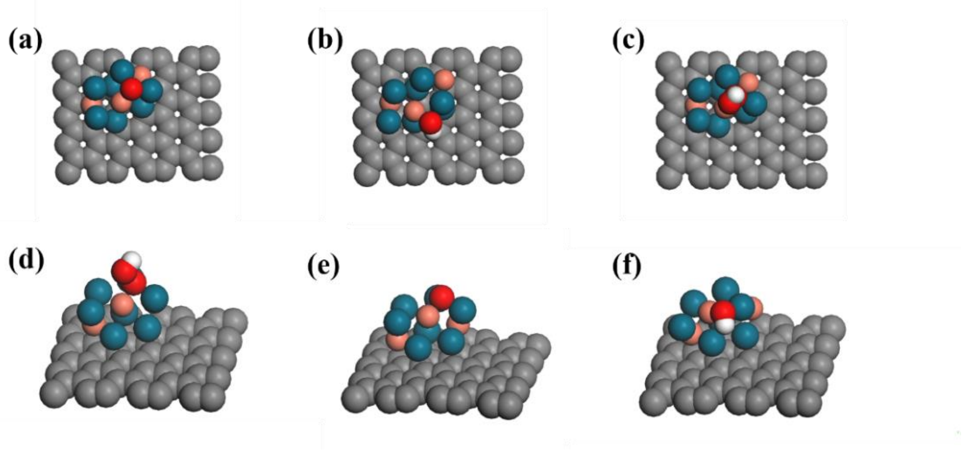

Figure S10 (a-c) Top view of site1 adsorption states OOH, O, and OH; (d-f) Main view of site5 adsorption states OOH, O, and OH.

Reference

1. Qin, Y.; Ou, Z.; Guo, C.; Liu, Y.; Jin, R.; Xu, C.; Chen, H.; Si, Y.; Li, H. Phosphor-doping modulates the d-band center of Fe atoms in Fe-N_4 catalytic sites to boost the activity of oxygen reduction. *Appl. Catal. B Environ.* **2025**, *360*, 124553.
2. Seong, H.; Min, K.; Lee, G.; Kwon, K.; Baeck, S.-H. Development of an efficient bifunctional electrocatalyst based on Co/$CoSe_2$ nanoparticles embedded in N, Se co-doped carbon for AEMFC and rechargeable Zn-air battery. *Appl. Catal. B Environ.* **2025**, *362* 124725.
3. Yin, X.; Xi, W.; Wang, P.; Wu, T.; Liu, P.; Gao, B.; Zheng, Y.; Lin, B. In-situ construction of 2D β-$Co(OH)_2$ nanosheets hybridized with 1D N-doped carbon nanotubes as efficient bifunctional electrocatalyst for oxygen reduction and evolution reactions. *Chem. Eng. J.* **2025**, *503* 158437.
4. Liang, Y.; Zhang, D.; Zhang, Y.; Yan, F.; Sun, L.; Jin, X.; Wang, Q.; Zheng, L.; Li, W. Regulation of the diatomic-site electronic structure with alloy nanoparticles in hollow carbon nanospheres for efficient oxygen reduction and evolution reactions. *Chem. Eng. J.* **2025**, *503* 158579.
5. Wu, X.; Ni, C.; Man, J.; Shen, X.; Cui, S.; Chen, X. A strategy to promote the ORR electrocatalytic activity by the novel engineering bunched three-dimensional Pd-Cu alloy aerogel. *Chem. Eng. J.* **2023**, *454*, 140293.
6. Zou, X.; Lu, Q.; Wu, J.; Zhang, K.; Tang, M.; Wu, B.; She, S.; Zhang, X.; Shao, Z.; An, L. Screening Spinel Oxide Supports for RuO_2 to Boost Bifunctional Electrocatalysts for Advanced Zn–Air Batteries. *Adv. Funct. Mater.* **2024**, *34*, 202401134.
7. Wang, M.; Xie, J.; Lu, Z.; Wang, J.; Yin, X.; Cao, Y. Biphase Alloy Nanoheterojunction Encapsulated within N-Doped Carbon Nanotubes as Bifunctional Oxygen Electrocatalyst for High-Performance Zn-Air and Mg-Air Batteries. *Adv. Funct. Mater.* **2025**, 202423767.
8. Li, Y.; Luo, X.; Wei, Z.; Zhang, F.; Sun, Z.; Deng, Z.; Zhan, Z.; Zhao, C.; Sun, Q.; Zhang, L. Precisely constructing charge-asymmetric dual-atom Fe sites supported on hollow porous carbon spheres for efficient oxygen reduction. *Energy Environ. Sci.* **2024**, *17*, 4646-4657.
9. Chen, Z.; Cheng, W.; Cao, K.; Jin, M.; Rahali, S.; Chala, S.A.; Ebrahimi, E.; Ma, N.; Liu, R.; Lakshmanan, K. A Bifunctional Iron-Nickel Oxygen Reduction/Oxygen Evolution Catalyst for High-Performance Rechargeable Zinc-Air Batteries. *Small.* **2024**, *21*, 202409161.
10. Shi, H.; Zhang, L.; Huang, X.; Kong, Q.; Abdukayum, A.; Zhou, Y.; Cheng, G.; Gao, S.; Hu, G. Efficient Catalysis for Zinc-Air Batteries by Multiwalled Carbon Nanotubes-Crosslinked Carbon Dodecahedra Embedded with Co-Fe Nanoparticles. *Small.* **2025**,202409129.
11. Zou, J.; Bao, L.; Sun, Q.; Bao, C.; Chen, H.; Liu, H. Oxygen Reduction Reaction Catalysts for Zinc-Air Batteries Featuring Single Cobalt Atoms in a Nitrogen-Doped 3D-Interconnected Porous Graphene Framework. *Small.* **2025**, 202409506.
12. Huang, S.; Tranca, D.; Rodríguez-Hernández, F.; Zhang, J.; Lu, C.; Zhu, J.; Liang, H.W.; Zhuang, X. Well-defined N_3C_1-anchored Single-Metal-Sites for Oxygen Reduction Reaction. *Angew. Chem. Int. Edit.* **2023**, *63*, e202314833.
13. Ban, J.; Wen, X.; Xu, H.; Wang, Z.; Liu, X.; Cao, G.; Shao, G.; Hu, J. Dual Evolution in Defect and Morphology of Single-Atom Dispersed Carbon Based Oxygen Electrocatalyst. *Adv. Funct. Mater.* **2021**, *31*, 2010472.
